# Primary malignant mesenchymoma of bladder: Case report and review of the literature

**DOI:** 10.1097/MD.0000000000007579

**Published:** 2017-08-11

**Authors:** Zecheng Yang, Xiaodong Yang, Xianqi Lu, Lijian Gao, Gang Li, Xuefeng Zhang

**Affiliations:** a Department of General Surgery, The Second Hospital of Jilin University, Changchun City, Jilin Province; b Department of Urology, Dezhou People Hospital, Dezhou City, Shandong Province; c Department of Urology, The Second Hospital of Tianjin Medical University, Tianjin Institute of Urology, Tianjin City; d Department of Urology, Weihai Central Hospital, Weihai City, Shandong Province, China.

**Keywords:** bladder tumors, malignant tumor, mesenchymoma

## Abstract

**Rationale:**

Malignant mesenchymoma (MM) is defined as a heterogeneous malignant soft tissue tumor that consists of 2 or more distinctly different mesenchymal components in addition to fibrosarcomatous elements. Bladder MM was rarely reported in the literature and there are only 5 cases of primary bladder MM documented in English literature to date.

**Patient concerns:**

A 58-year-old male complained of difficulty in urination and intermittent gross hematuria for 3 months. Doppler ultrasound scan revealed an avascular and homogeneous hypoechoic mass measured 6.5 × 9 cm in the bladder. Computed tomography showed a homogeneous solid mass in the bladder.

**Diagnoses:**

Pathology revealed spindle-shaped tumor and proliferation of poorly differentiated immature mesenchymal cells rich in eosinophilic cytoplasm with hyperchromatic sticklike nuclei. Immunohistochemical examinations were positive for CD117.

**Interventions:**

The patient was diagnosed with presence of bladder tumor and underwent radical cystectomy; the optimal treatment strategy was reviewed and discussed.

**Outcomes:**

There was no recurrence or metastasis during a 16-month follow-up.

**Lessons:**

Our case study demonstrated bladder MM with a relatively indolent clinical course. A multidisciplinary approach including surgery, radiotherapy, and chemotherapy may be useful. Radical resection is the most important determinant of clinical outcome. Generally, the clinical outcome and prognosis of mesenchymoma are favorable.

## 1 Introduction

Malignant mesenchymoma (MM) is a rare tumor and was first described by Stout in 1948^[1]^; the histogenesis and etiology were uncertain. It was defined as a malignant soft tissue tumor that consists of 2 or more distinctly different mesenchymal components. Primary MM of bladder was reported occasionally; a review of the literature revealed only 5 cases fit the criteria of primary bladder MM.^[2–6]^ To our knowledge, the present case is the sixth case in the English literature to date.

## 2 Case report

**Figure 1:**
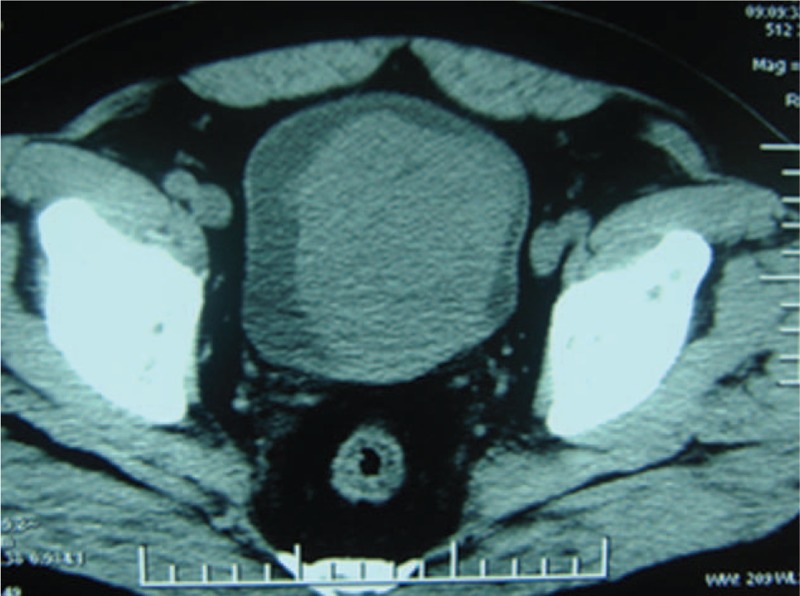
Abdominal CT shows a solid mass with relatively low density in comparison to the adjacent muscles in the bladder.

**Figure 2:**
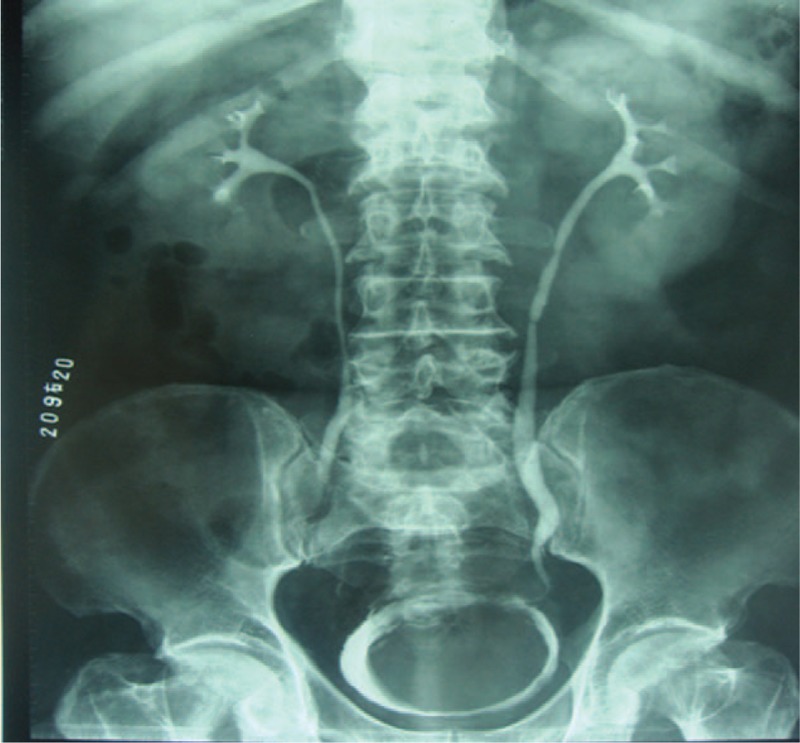
IVP indicated a huge filling defect in the areas of bladder. IVP = intravenous pyelogram.

The patient was a 58-year-old male who complained of difficulty in urination and intermittent gross hematuria for 3 months. He was admitted to our hospital on June 1, 2010. There was no family history of cancer or chronic illness. Rectal examination disclosed a mildly enlarged mass just like benign prostate. Abdominal exploration was unremarkable. Urinalysis revealed gross and microscopic hematuria though urine Gram stain did not suggest infection. Biochemical investigations revealed mild hyperbilirubinemia and the rest parameters were normal. Urinary cytology studies showed no abnormalities. Laboratory findings showed blood counts, electrolytes, and other routine studies were all normal. The preoperative Doppler ultrasound scan revealed an avascular and homogeneous hypoechoic mass measuring 6.5 × 9 cm in the bladder. A subsequent computed tomography (CT) demonstrated a large mass showing homogeneous density in the bladder region without obvious enlarged lymph nodes (Fig. 1). Intravenous pyelogram (IVP) indicated a huge filling defect in the areas of the bladder (Fig. 2) though it was normal in the upper urinary tract. At cystoscopy, a nonpapillary and nonpedunculated tumor was seen on the trigone wall of the bladder. The lesions occupied almost the entire vesical cavity. Two biopsy specimens were sampled separately for histological examination. Autopsy specimen showed immature mesenchymal cells with fibrous change and differentiated lesion composed predominantly of spindle-shaped cells. It suggested to the possibility of a mesenchymoma with immature differentiation tendency.

**Figure 3:**
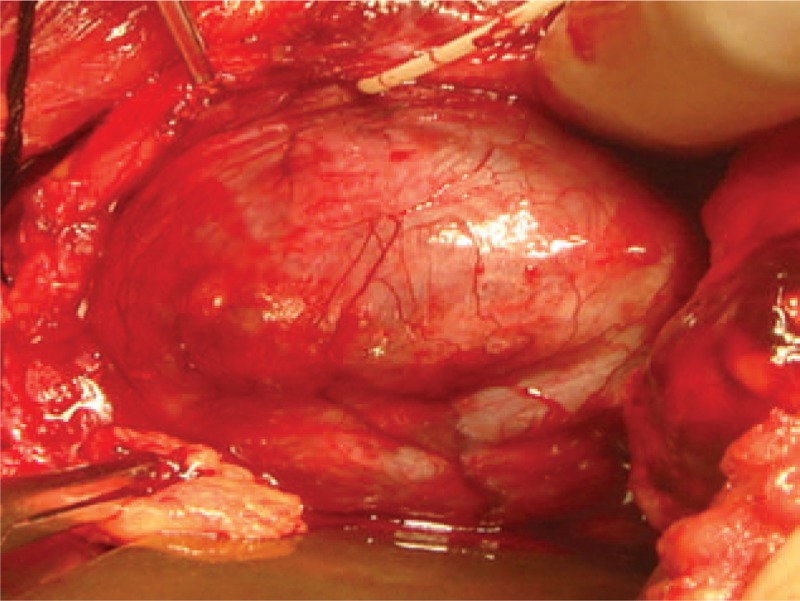
On operating, a well-circumscribed tumor measuring about 9 × 7 cm was found with a relatively large pedicle.

Based on the patient's clinical history and the biopsy results, unspecified malignancy or benign mesenchymal origin was revealed. It appeared the best to proceed with open surgical partial cystectomy under general anesthesia. Before operation, the chest x-ray and ultrasound scan of the retroperitoneum and abdomen did not reveal signs of tumor dissemination. On exploration through a suprapubic approach a distended bladder was revealed; a well-circumscribed tumor about 10 cm in diameter was found in the trigone of bladder with relatively large pedicle parts (Fig. 3). It was hard to find the left ureter orifice. There was no significant abnormality in the adjacent structures. Partial cystectomy would induce insufficient bladder volume. Complete resection of the tumor was impossible at that time so we decided to perform radical cystectomy and urinary diversion of bilateral ureterocutaneostomy. The postoperation and convalescence went smoothly.

**Figure 4:**
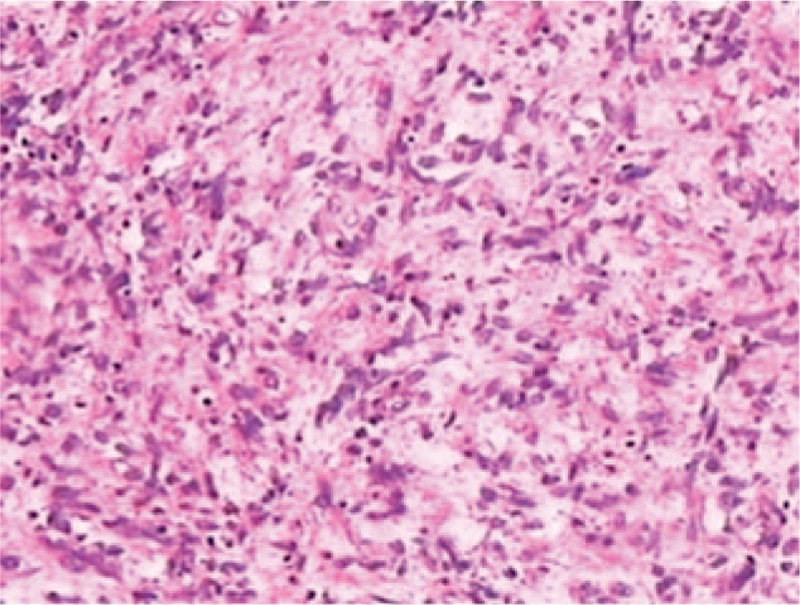
Microscopically, the tumor cells were composed of pleomorphic spindle tumor cells and rich in eosinophilic cytoplasm. Proliferation of poorly differentiated immature mesenchymal cells with hyperchromatic sticklike nuclei, which are minimal atypia, was seen (hematoxylin and eosin staining, ×200).

**Figure 5:**
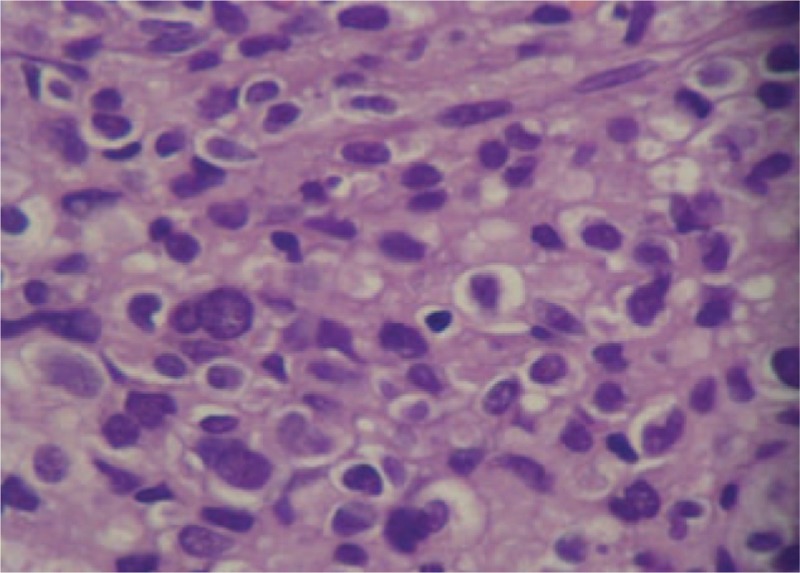
Immature mesenchymal cells with hyperchromatic sticklike and atypia nuclei (hematoxylin and eosin staining, ×400).

**Figure 6:**
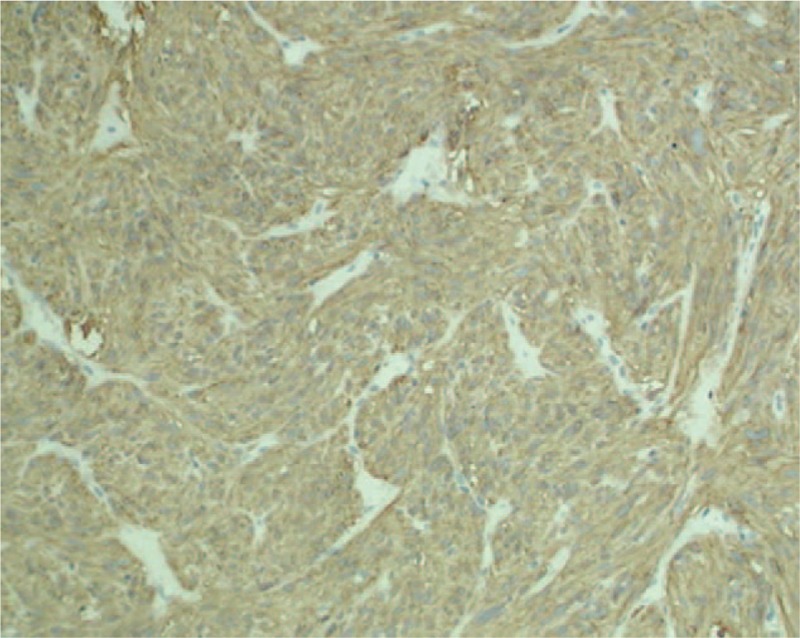
An immunohistochemical examination shows focally positive immunoreactivity of CD117 (immunohistochemical staining, ×100).

Gross pathologic examination showed a mass measuring 6 × 7 × 9 cm encapsulated by smooth vesical mucosa and containing myxoid areas. The cut surfaces had a homogeneous, grayish-white appearance without necrosis. Tumor tissue was fixed in 10% formaldehyde neutral and embedded in paraffin according to conventional procedures. Histologically, the tumor was composed of fiber and had bundle-shaped structure. The tumor cells were in spindle shape and rich in eosinophilic cytoplasm (Fig. 4). Proliferation of poorly differentiated immature mesenchymal cells with hyperchromatic sticklike nuclei which are minimal atypia could be seen. The mitotic figure was higher than 5 mitoses per 50 high-power fields (Fig. 5). The tumor had superficial muscular layer invasion, and immunohistochemical examinations demonstrated it positive for CD117 (Fig. 6) whereas negative for Desmin, SMA, CK, and CD34. The final histological diagnosis was primary MM of urinary bladder. The dominant architectural pattern of the tumor was of spindle cells in a storiform arrangement, resembling a so-called malignant fibrous histiocytoma. Cellular polymorphism was marked and coupled with numerous typical and atypical mitotic figures.

The patient did not receive any adjuvant chemotherapy, and with a follow-up for 16 months after surgical operation, he was found free of the disease without recurrence and metastasis on routine examination.

## 3 Discussion

MM arises from a primitive mesenchymal cell with the capacity for totipotent differentiation. It can occur at all locations in the body, especially in the retroperitoneum, soft tissue, and digestive tract such as gastrointestinal stromal tumors (GIST), which are predominant in adults or malignant extra-GIST, which are very rare.^[7]^ The disease usually presents with abdominal pain, hematuria, dysuria, or a palpable mass but with distant metastasis in some patients. In radiological terms, most MMs exhibited solid mass with larger size than that of common form of the bladder cancer, just as our case, presenting with homogeneous density in comparison with the adjacent muscles. MM may demonstrate various radiologic features. Calcification or necrosis was visible within the tumor whereas enlarged lymph nodes were rare. There was little literature to describe the radiological character of MM. The radiologic findings of well-circumscribed and heterogeneous mass which, including osteosarcomatous components, were considered to be MMs.^[8]^ In our case, tumor was a homogeneous mass and without calcification. It was hard to determine malignant character although the biopsy was done before operation. The correct diagnosis needed immunohistochemical examinations, especially CD117 and CD34.^[9]^ It is difficult to make the diagnosis of MM by radiological imaging characters which were often unspecific. Imaging and clinical features are of no great help to the diagnosis of MM.^[10]^ The differential diagnosis must take into account primary or metastatic tumors (especially musculoskeletal tumors), as well as mesothelioma and other rare sarcomas. There is corresponding relationship between the size of the tumor and cells atypia and the prognosis of MM. Immunohistochemical studies were necessary to establish the diagnosis.

Given its scarcity, there are insufficient data to suggest the optimal management strategy for MM of the urinary bladder. A multidisciplinary approach including surgery, radiotherapy, and chemotherapy may be useful. Radical resection is the most important determinant of clinical outcome. Chemotherapy and radiotherapy were ineffective for the soft parts of the sarcoma,^[11]^ including the MM. Radiology and/or chemotherapy resulted in a differential response. Randomized clinical trial illustrated that targeted treatments such as high-dose Imatinib can improve progression-free survival in gastrointestinal stromal tumors.^[12]^ In our case, the clinical course was rather indolent. Although without any adjuvant chemotherapy or radiotherapy, there was no evidence of metastasis during the subsequent 16-month period. The patient was in good health; it seems to be a more favorable prognosis.

In the 3 available documented bladder MMs, sizes were 18 × 10 × 9, 14 × 13 × 7, and 14 × 13 × 7 cm, respectively. It seems to be larger than traditional bladder transitional carcinoma (BTCC). Among these 3 cases, only 2 have detailed follow-up data. One patient received surgery, chemotherapy, and irradiation, and had a total life of 21 months.^[13]^ The other just received radical cystectomy and died 2 months after diagnosis. The prognosis of MM is poor. With few exceptions, the median survival time is about 30 months from diagnosis.^[14]^

## 4 Conclusion

The prognosis of MM remains controversial. There is no statistically significant prognosis indicator with respect to gender, tumor site, tumor size, or MIB-1-labeling index.^[15]^ Patients less than 40 years old or those who had an RMS component showed significantly worse prognosis; the other mesenchymal components showed no significant correlation with survival.
